# Beyond Domain-Specific Expertise: Neural Signatures of Face and Spatial Working Memory in Baduk (Go Game) Experts

**DOI:** 10.3389/fnhum.2018.00319

**Published:** 2018-08-07

**Authors:** Wi Hoon Jung, Tae Young Lee, Youngwoo B. Yoon, Chi-Hoon Choi, Jun Soo Kwon

**Affiliations:** ^1^Department of Psychology, Korea University, Seoul, South Korea; ^2^Institute of Human Behavioral Medicine, Medical Research Center, Seoul National University, Seoul, South Korea; ^3^Brain and Cognitive Sciences, College of Natural Sciences, Seoul National University, Seoul, South Korea; ^4^Department of Psychiatry, Washington University in St. Louis, St. Louis, MO, United States; ^5^Department of Radiology, Chungbuk National University Hospital, Cheongju, South Korea; ^6^Department of Psychiatry, College of Medicine, Seoul National University, Seoul, South Korea

**Keywords:** board game, connectome, frontoparietal network, functional connectivity, holistic processing

## Abstract

Recent advances of neuroimaging methodology and artificial intelligence have resulted in renewed interest in board games like chess and Baduk (called Go game in the West) and have provided clues as to the mechanisms behind the games. However, an interesting question that remains to be answered is whether the board game expertise as one of cognitive skills goes beyond just being good at the trained game and how it maps on networks associated with cognitive abilities that are not directly trained. To address this issue, we examined functional activity and connectivity in Baduk experts, compared to novices, while performing a visual n-back working memory (WM) task. We found that experts, compared to novices, had greater activation in superior parietal cortex during face WM, though there were no group differences in behavioral performances. Using a data-driven, whole-brain multivariate approach, we also found significant group differences in the multivariate pattern of connectivity in frontal pole and inferior parietal cortex, further showing greater connectivity between frontal and parietal regions and between frontal and temporal regions in experts. Our findings suggest that long-term trained Baduk experts have the reorganization of functional interactions between brain regions even for untrained cognitive ability.

## Introduction

People have very different levels of cognitive ability, from profound impairments to superior skills. However, while our understanding of neural mechanisms of cognitive impairments has been greatly enhanced via neuroimaging studies for general and cognitive-impaired individuals, those of superior skills still remain poorly understood.

**FIGURE 1:**
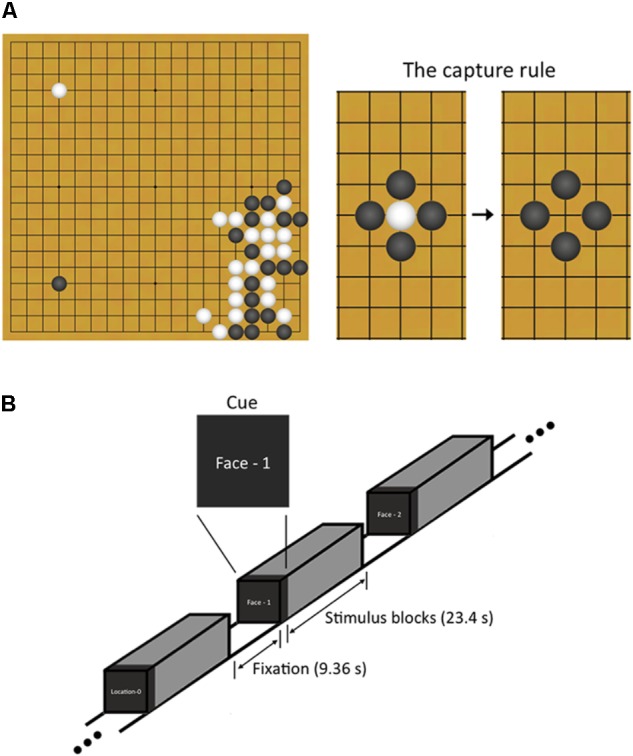
Explanation about Baduk and n-back working memory task used in this study. **(A)** Baduk is played using black and white stones on a square board with a 19 by 19 grid of lines. The object of Baduk is to surround and capture more empty territory on the board than the opponent but not to surround and capture the opponent’s stones. However, one strategy to achieve this object is to surround the opponent’s stones because all captured stones are removed from the board and the areas where the stones were removed from become the territory of the capturing player. **(B)** During brain scanning, participants performed a block-designed n-back task including both face and spatial working memory conditions. Each block included 10 trials (23.4 s) and was split up with the resting blocks (9.36 s). Visual instructions (2.34 s) preceded each block to indicate the upcoming condition.

Baduk, as it is called in Korea (known as the game of Go in the West^1^), is an abstract strategy board game like chess; it is played on a square board with a 19 by 19 grid of lines. Its rule is simple; two players, one playing with white stones and the other playing with black ones, take turns placing a stone to capture more territory on the board than the opponent by surrounding the opponent’s stones (**Figure 1A**). Despite such simple rule, Baduk is considered more complex than chess owing to its enormous branching factors (i.e., the enormous number of move choices available) (Keene and Levy, 1991).

Board games like Baduk and chess have been commonly used to investigate the mechanism underlying cognitive expertise, as playing involves diverse high-level cognitive functions such as visuospatial processing, decision making, and attention (Chase and Simon, 1973b; Gobet and Charness, 2006). For example, decades of neuroimaging studies have identified multiple brain regions engaged during board game play, including dorsolateral prefrontal cortex, premotor cortex, and occipitotemporal and parietal cortices (Chen et al., 2003; Atherton et al., 2003; Itoh et al., 2008). Additionally, researchers have explicitly investigated neural correlates of specific cognitive components closely related to board game by using a variety of domain-specific tasks employing the board game-related stimuli; these studies have revealed brain regions associated with object and pattern recognition on the board, such as the occipitotemporal junction, fusiform gyrus (FFA), and collateral sulcus (Bilalić et al., 2010, 2011), with intuitive best next-move generation on the board, such as the caudate nucleus (Wan et al., 2011, 2012), and with intuitive strategy decision making on the board, such as different parts of the cingulate cortex (Wan et al., 2015). Some of these regions, particularly in the occipitotemporal junction, FFA, and caudate nucleus, also showed significant group differences in structural morphology as well as structural and functional connectivity (FC) at rest between board game experts (BEs) and novices (Lee et al., 2010; Duan et al., 2012a,b, 2014; Jung et al., 2013; Hänggi et al., 2014). Interestingly, a recent meta-analysis on functional neuroimaging studies of long-term expertise has suggested the common mechanisms across different cognitive domains, showing enhanced or additional activity in the brain of experts compared to novices (Neumann et al., 2016). Especially, the meta-analysis on the studies with visual stimulation showed that experts had enhanced activation in inferior parietal cortex and lingual gyrus.

Despite the efforts mentioned above, an interesting question that remains to be answered is whether the board game expertise goes beyond just being good at the trained game. In other words, do behavioral and neural differences between BEs and novices exist in untrained cognitive abilities? Although there are some studies investigating the effects of chess instructions on untrained tasks (Sala and Gobet, 2016), it is still unclear whether board game expertise transfers to other cognitive skills. Moreover, most of functional neuroimaging studies comparing BEs and novices so far have focused on the regional areas associated with visual expertise using both trained and untrained stimuli (e.g., chess-related objects and positions, faces, rooms, and tools), such as FFA and occipitotemporal and occipitoparietal areas (Bilalić et al., 2010, 2011; Bilaliæ, 2016), rather than functional networks associated with other specific cognitive abilities.

Working memory (WM) is involved in the frontoparietal network (Owen et al., 2005). Particularly, object and spatial WM may be one of cognitive abilities that can be potentially enhanced through board game training based on its important role in the game; in the case of Baduk, to win the game, players are needed to remember the positions of stones on the board and to hold several future offensive moves and the opponent’s expected responses to each of the future moves in their WM. There have been existed several studies to test brain activity during 1-back WM task in chess experts compared to novices using chessboards and faces or scenes stimuli (Bilalić et al., 2011; Krawczyk et al., 2011; Bartlett et al., 2013). These studies reported consistent behavioral results, showing no group differences in behavioral performances except board-specific stimuli, but inconsistent neural results, showing an increase in BEs (Bilalić et al., 2011) or no group difference in FFA activation in response to chessboards (Krawczyk et al., 2011). These previous studies using 1-back task have investigated the neural activity in response to the recognition of trained and untrained stimuli rather than WM in BEs. Additionally, there is no a study to test functional coupling between brain regions during WM in BEs.

Here we examined the functional activity and connectivity of Baduk experts, compared to novices, while performing a visual n-back WM task with both object and spatial WM conditions. Especially, we chose to use neutral face stimuli for the task because like the board games, face discrimination and recognition rely on at least partly on holistic processing and reliably activate the FFA (Kanwisher et al., 1997), where the region is a general visual expertise module rather than face-specific (Gauthier et al., 1999, 2000). That is, using face stimuli allows us to test whether there are group differences in FFA function (Bilalić et al., 2011; Krawczyk et al., 2011; Bartlett et al., 2013). We also used a recently introduced FC technique, called multivariate distance-based matrix regression (MDMR) as part of a connectome-wise association study (CWAS) to identify brain regions showing group differences in the connectome, whole-brain FC patterns (Shehzad et al., 2014). It is a fully data-driven, whole-brain multivariate analytic approach, which provides a more comprehensive characterization of brain-behavior relationships than massive univariate approach. In the present study, the MDMR-based CWAS allowed us to evaluate the overall multivariate patterns of FC associated with a phenotype (BEs vs. novices as group) at each voxel while controlling confounding variables (age, sex, and in-scanner motion). Based on previous findings, we hypothesized that BEs, compared to novices, showed increased functional activity and connectivity particularly in the frontoparietal network during the n-back WM task.

## Materials and Methods

### Participants

Seventeen BEs participating in Baduk training from their childhood were recruited from the Korea Baduk Association^2^. Seventeen novices who were age, sex, and IQ-matched with those in the BEs, also recruited through online advertisements for purpose of comparison. Based on the simple screening questionnaire, all participants were not experts in any games, except Baduk for BEs. All participants were also right-handed and had no history of psychiatric or neurological disease. This dataset included resting-state fMRI, n-back WM task-based fMRI, T1-weighted anatomical MRI, and diffusion tensor imaging. Previous reports from this dataset have concerned differences in anatomical connectivity (Lee et al., 2010) and in gray matter volume (GMV) and resting-state FC (Jung et al., 2013) between BEs and novices. The procedures of this study were approved by the Institutional Review Board of Seoul National University Hospital and written informed consent was obtained from all participants, including parental consent for those younger than 18 years of age. All methods were performed in accordance with the approved guidelines and regulations.

Here we analyzed fMRI data obtained during the n-back WM task. Four participants (2 BEs and 2 novices) out of 34 were excluded from analyses due to excessive missing trials during the task. The final sample consisted of 15 BEs (mean age 17.3 years, range 16–20 years) and 15 novices (mean age 17.0 years, range 15–19 years; mean training duration 12.6 years). Our sample size corresponded to samples used in previous behavioral (Bilalić et al., 2008, 2009) and neuroimaging studies with BEs (Bilalić et al., 2011; Bilaliæ, 2016). Despite even the relatively small sample sizes, the direct comparison between experts and novices can provide power to capture the effects of interests.

### Task

Participants performed a block-designed n-back WM task including both face matching and spatial location matching conditions (**Figure 1B**). The task had four WM loading conditions: 0-back, 1-back, 2-back, and 3-back. The 0-back served as a control condition, in which participants responded with a button press to a predetermined target stimulus. For the n-back face matching WM conditions, participants responded if the current face was identical to the previous one, two, and three faces ago, respectively. For the n-back spatial location matching WM conditions, participants responded if the current face was in the same place n faces ago regardless of the face identity. During the task, participants responded by pressing a button with their right index finger. The face stimuli consisted of 20 gray-scale pictures of neutral faces (10 Korean men and 10 Korean women), selected from stimuli used in our previous studies (Shin et al., 2008, 2015). The stimuli appeared in 35 different spatial positions on the screen during both the face identity and spatial tasks.

The task consisted of four runs with 48 blocks (12 blocks per run), resulting in six blocks for each of four loading conditions on face and spatial WM. Blocks were presented pseudo-randomly in order of increasing (or decreasing) memory load. Each block included 10 trials (23.4 s) and was split up with the resting blocks of 4 TRs (9.36 s). Visual instructions (2.34 s) preceded each block to indicate the upcoming condition. A face stimulus for each trial was presented for 1500 ms followed by 840 ms of fixation. Before scanning, the participant was given practice to learn the task rules.

### Image Acquisition

All image data were acquired on a 1.5T Siemens Avanto MRI scanner. While performing the task, fMRI data were obtained via a gradient echo-planar image pulse sequence (repetition time (TR)/echo time (TE) = 2340/52 ms, flip angle (FA) = 90°, field of view (FOV) = 220 mm, voxel size = 3.44 mm × 3.44 mm × 5 mm). High-resolution anatomical images were acquired with T1-weighted 3-D MPRAGE sequence (TR/TE = 1160/4.76 ms, FA = 15°, FOV = 230, matrix size = 256 × 256). Other image parameters that are not related to the present study are not described herein.

### Conventional fMRI Analysis

Image analysis was performed with SPM12^3^. For each participant, after discarding the first three images in each run, images were then corrected for slice timing, realigned to the first volume, and co-registered with each participant’s structural image. There were no significant group differences in the head motion parameters. All images were then normalized to the MNI space using the normalization parameters estimated from the structural MRI. The normalized images were smoothed with 6 mm FWHM Gaussian kernel. First level-analyses were performed using the general linear model (GLM) with regressors for the cue, 0-back, l-back, 2-back, and 3-back conditions for each type of face and spatial WM in addition to six head motion parameters and a constant term. The regressors were modeled as boxcar functions convolved with a canonical hemodynamic response function for the length of each condition. To delineate brain regions activated during WM for each group and determine regions showing group differences, one-sample *t*-tests and two-sample *t*-tests were, respectively, performed for the contrast images of the 1-, 2-, 3-back versus 0-back conditions for each type of face and spatial WM. To further explore a group by WM load interaction for each type of face and spatial WM, we also performed a two (group) by three (WM load) analysis of variance (ANOVA) using the following contrast images: 1-back > 0-back, 2-back > 0-back, and 3-back > 0-back. The results were corrected for multiple comparisons to a significance level of *p* < 0.05 (height threshold of *p* < 0.001, uncorrected, combined with extent threshold of *p* < 0.05, family-wise error [FWE]-corrected).

### MDMR-Based CWAS Analysis

The MDMR-based CWAS method has been described in detail elsewhere (Shehzad et al., 2014; Satterthwaite et al., 2015). For MDMR-based CWAS analysis, the preprocessed image data were down-sampled to 4-mm isotropic voxels for the purpose of computational feasibility. Next, the first two data-points (4.68 s) from every task block were excluded to account for the hemodynamic delay and next all volumes of the same task type (i.e., face or spatial n-back WM) were concatenated across load levels, except for 0-back, for each participant. We then performed the MDMR-based CWAS analysis according to the following three steps using Connector, an R package for CWAS^4^. First, we computed FC (Pearson correlation coefficient) between time series of a given voxel and those of every other voxel within the gray matter mask including cortical and subcortical areas. Second, we evaluated the overall multivariate pattern of FC for a given voxel by calculating a distance metric between every pair of FC calculated above for a given voxel (e.g., between two participants’ FC for a given voxel). To calculate the distance metric, we used $\sqrt{2(1-\gamma )}$, where γ is the Pearson correlation, resulting in a non-negative value that indicates how similar/different each pair is (0 = perfectly correlated, 2 = perfectly negatively correlated). Third, MDMR was used to test how well a phenotypic variable explains the distances between participants calculated in the second step. To examine group differences in FC between BEs and novices while controlling for confounding variables, modeled variables included group (BEs versus novices), age, sex, and head motion indexed by mean framewise displacement (Power et al., 2012). For each voxel’s FC pattern, MDMR yielded a pseudo-F statistic from a standard ANOVA model, by comparing the sum of squared distances between BEs and novices to the residual sum of squared distances (the error term), whose significance (i.e., *p*-value) was assessed using 5,000 iterations of a permutation test. All these steps were repeated for every gray matter voxel to produce a whole-brain *p*-value map. The *p*-value map was converted to *z*-value for multiple comparisons corrections. As in Satterthwaite et al. (2016), the z-map was thresholded at a voxel height of *z* > 2.326 and a corrected probability of *p* < 0.05 using 10,000 Monte Carlo simulations.

### Follow-Up Seed-Based Connectivity Analysis

Although MDMR-based CWAS identifies clusters where group differences are present based on multivariate patterns of FC, it does not provide specific connections and direction of observed clusters (Shehzad et al., 2014). Accordingly, we performed *post hoc* seed-based FC analyses for each cluster identified by MDMR-based CWAS. Seed-based FC maps were generated by Pearson correlation between time series of each seed cluster and those of every other voxel and then Fisher r-to-z transformed. Two-sample *t*-tests were conducted to examine group differences in z-transformed seed-based FC maps. Statistical significance was set at a voxel-level FWE-corrected *p* < 0.05.

### Region-of-Interest (ROI) Analysis

Once regions showing significant effects from the aforementioned analyses were detected, we further conducted partial correlation analyses (controlling for age and sex) between brain measures (i.e., neural activity measured as the percent signal change extracted by MarsBar toolbox^5^ or the strength of FC) in the identified regions as functional ROI and behavioral performances during the task and training duration in BEs.

## Results

### Demographic and Behavioral Data

**Table 1** summarizes demographic information and behavioral performances during the n-back WM task in both BEs and novices. There were no significant group differences in age, sex, education, and IQ (all *p*s > 0.05). The two groups did not differ significantly in the accuracy and the reaction time (RT) during the face and spatial n-back WM (all *p*s > 0.05).

**Table 1:** Demographic and behavioral performance measures in both novices and Baduk experts.

Variables	Experts	Novices	*t*-value	*p*-value
**Demographic data**				
Age (years)	17.33 (1.11)	17.00 (1.13)	0.813	0.423
Full scale IQ	94.00 (10.37)	100.53 (12.91)	–1.528	0.138
Education (years)	10.40 (1.30)	10.73 (1.33)	–0.6934	0.693
Sex (male/female)	11/4	12/3	0.186^a^	0.666
Training duration (years)	12.60 (1.55)	–	–	–
**Behavioral data during face n-back**				
0-back accuracy (% correct)	95.93 (9.26)	97.41 (8.62)	–0.454	0.654
1-back accuracy (% correct)	89.82 (9.64)	90.88 (9.84)	–0.296	0.769
2-back accuracy (% correct)	78.67 (17.88)	76.33 (16.63)	0.37	0.714
3-back accuracy (% correct)	61.11 (19.58)	67.41 (17.80)	–0.921	0.365
Overall accuracy (% correct)^b^	76.53 (13.67)	78.21 (12.61)	–0.349	0.73
0-back reaction time (ms)	665 (86)	615 (63)	1.788	0.085
1-back reaction time (ms)	849 (108)	805 (83)	1.233	0.228
2-back reaction time (ms)	917 (107)	899 (116)	0.437	0.665
3-back reaction time (ms)	991 (110)	960 (119)	0.759	0.454
Overall reaction time (ms)^b^	919 (99)	888 (89)	0.9	0.376
**Behavioral data during spatial n-back**				
0-back accuracy (% correct)	97.78 (4.60)	97.78 (4.60)	0	1
1-back accuracy (% correct)	96.14 (10.04)	96.49 (5.14)	–0.12	0.905
2-back accuracy (% correct)	95.19 (10.88)	90.00 (10.95)	1.301	0.204
3-back accuracy (% correct)	87.45 (12.35)	83.92 (17.15)	0.647	0.523
Overall accuracy (% correct)^b^	92.93 (10.38)	90.14 (8.41)	0.808	0.426
0-back reaction time (ms)	564 (116)	516 (95)	1.236	0.227
1-back reaction time (ms)	652 (135)	598 (98)	1.256	0.219
2-back reaction time (ms)	706 (142)	677 (117)	0.613	0.545
3-back reaction time (ms)	805 (176)	720 (202)	1.227	0.23
Overall reaction time (ms)^b^	721 (138)	665 (124)	1.17	0.252

*Values in this table are presented as mean (standard deviation). Independent *t*-tests were used for statistical analyses of all variables except sex (male or female). ^*a*^A chi-square test was used. ^*b*^Overall accuracies and reaction times were estimated across 1-, 2-, and 3-back conditions for each type of face and spatial n-back. IQ, intelligence quotient. Participants’ IQs were estimated by Korean–Wechsler Adult Intelligence Scale-Revised (K-WAIS-R).*

### Group Differences in Neural Activity

**FIGURE 2:**
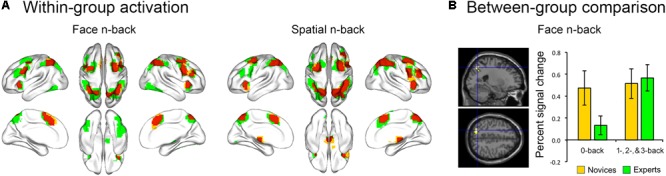
Brain activation during the n-back task. **(A)** The overlap (red) of activation maps for experts (green) and novices (yellow) during each WM task. The frontal and parietal areas were activated during both face and spatial n-back across working memory load (the 1-, 2-, and 3-back versus 0-back conditions). These activation maps were visualized with the BrainNet Viewer toolbox (Xia et al., 2013). **(B)** When comparing activation maps between experts and novices, experts showed greater activation in left superior parietal cortex (SPC) for the contrast of the face working memory load (1-, 2-, and 3-back) conditions versus the 0-back control condition. Percent signal change (PSC) was extracted for each participant and condition using MarsBar toolbox (http://marsbar.sourceforge.net/). The effect size was calculated by Cohen’s d to provide the standardized mean difference between the two groups, independent of sample size. The plot of mean PSC shows that group difference in the SPC region is caused by less activation in experts compared to novices for the 0-back condition (Cohen’s *d* = –0.694), whereas the PSCs of the working memory load condition for two groups are similar (Cohen’s *d* = 0.107).

Both BEs and novices showed similar activation patterns in the frontal and parietal regions during both face and spatial n-back WM (**Figure 2A** and **Table 2**). However, between-group comparisons revealed greater activation in the left superior parietal cortex (SPC) in BEs than novices during face WM at cluster-level family-wise error (FWE)-corrected *p* < 0.05 (**Figure 2B** and **Table 2**). The *post hoc* ROI analysis to further characterize the group difference in the region showed less SPC activation in BEs for the face 0-back control condition (*t*-/*p*-value = -1.904/0.067; Cohen’s *d* = -0.694), albeit only marginally significant, than novices but not for the face WM loading condition (*t*-/*p*-value = 0.292/0.773; Cohen’s *d* = 0.107). No significant correlations were found between neural activities in the SPC and behavioral performances (accuracy and RT) during the task and training durations in BEs (all *p*s > 0.05). There were no significant group differences in any other regions and other task conditions and no group by WM load interactions (all *p*s > 0.05).

**Table 2:** Brain regions significantly activated in experts and novices during working memory tasks and the cluster with a significant difference between groups.

	Experts		Novices		Experts vs. Novices	
Area	MRI coordinates	*t*-/*z*-values	MRI coordinates	*t*-/*z*-values	MRI coordinates	*t*-/*z*-values
**Face n-back task**					
Superior frontal cortex	–24, –1, 47	9.44/5.21				
Middle frontal cortex	39, 8, 26	7.12/4.56	–42, 11, 29	5.67/4.02		
Inferior frontal cortex	45, 32, 26	5.87/4.11	45, 11, 29	8.68/5.02		
Supplementary motor area	–9, 23, 38	6.75/4.43	0, 14, 53	8.86/5.06		
Insular cortex	33, 26, –1	8.20/4.89	–30, 23, –1	6.16/4.22		
	–27, 23, –7	7.45/4.66	33, 17, –4	5.98/4.15		
SPC/IPC	–27, –52, 38	9.15/5.14	–30, –55, 41	7.50/4.68	–24, –67, 47	5.22/4.33
Midbrain/Thalamus	6, –28, –22	5.82/4.08				
Fusiform gyrus	45, –40, –25	8.71/5.02				
	–45, –55, –16	7.25/4.60				
Cerebellum	–9, –52, –22	7.42/4.65	–12, –76, –25	5.66/4.02		
**Spatial n-back task**					
Middle frontal cortex	30, 11, 56	9.12/5.13	–24, –1, 50	8.26/4.90		
	36, 47, 20	6.76/4.44	33, 35, 32	7.76/4.76		
	–45, 32, 32	4.99/3.72	30, –1, 53	8.39/4.94		
Inferior frontal cortex	–48, 8, 29	6.96/4.50				
	51, 8, 26	7.63/4.72				
Supplementary motor area	0, 20, 50	12.12/5.76	3, 14, 53	8.04/4.84		
Insular cortex	30, 23,5	7.66/4.73	–33, 17, –10	8.33/4.92		
	–27, 26, –1	7.14/4.57	33, 20, 2	9.51/5.23		
SPC			–15, –64, 56	9.40/5.20		
IPC	–36, –46, 44	10.42/5.43	42, –43, 53	12.41/5.81		
Precuneus	6, –70, 47	9.12/5.13				
Thalamus/Midbrain	6, –25, –7	8.70/5.02	–9, –13, –4	6.19/4.23		
			9, –1, –1	6.18/4.23		
Cerebellum	–30, –61, –28	6.39/4.30	–27, –58, –31	9.55/5.24		
	–6, –73, –22	5.86/4.10	–9, –73, –22	5.70/4.04		

*All results presented at height threshold *p* < 0.001, uncorrected, and cluster-extent threshold *p* < 0.05, family-wise error corrected. For each region of activation, MNI (x, y, z) coordinates and *t*-/*z*-values are given in reference to the maximally activated voxel within each cluster. SPC, superior parietal cortex; IPC, inferior parietal cortex; PFC, prefrontal cortex.*

### Brain Regions Identified by CWAS

**FIGURE 3:**
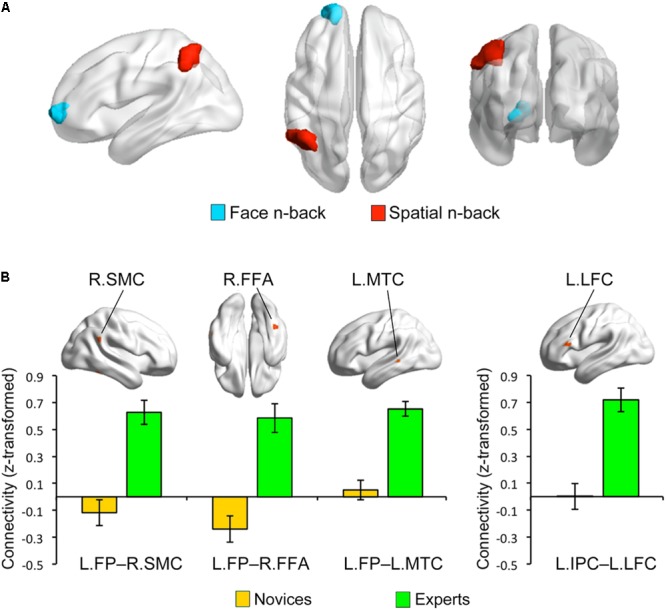
Group differences in functional connectivity. **(A)** Based on the multivariate patterns of functional connectivity, the left frontal pole (LFP) and inferior parietal cortex (LIPC) showed significant group differences during face and spatial n-back, respectively. **(B)** The seed-based connectivity analyses revealed that experts compared to novices had greater connectivity between the left frontal pole seed and right supramarginal cortex (RSMC) (Cohen’s *d* = 1.444), right fusiform cortex (RFFA) (Cohen’s *d* = 2.084), and left middle temporal cortex (LMTC) (Cohen’s *d* = 2.052) during the face n-back and greater connectivity between the left inferior parietal cortex seed and left lateral frontal cortex (LLFC) (Cohen’s *d* = 2.887) during the spatial n-back. Note that these bar graphs are presented for visualization purposes only.

Multivariate distance-based matrix regression-based CWAS analyses revealed two regions where the multivariate patterns of FC differ between BEs and novices at cluster-level corrected *p* < 0.05; one is the left frontal pole (FP; peak MNI *x, y, z* coordinates = -16, 60, 0) for face WM condition and the other is the left inferior parietal cortex (IPC; *x, y, z* = -52, -52, 44) for spatial WM condition (**Figure 3A**).

### Group Differences in Seed-to-Voxel Connectivity

To further characterize the FC of the regions identified by MDMR-based CWAS, we performed *post hoc* seed-based FC analysis to each of these identified clusters (**Figure 3B**). For face WM condition, BEs compared to novices had greater FC between the left FP seed and right FFA (*x, y, z* = 44, -48, -20; *t*-/*z*-values = 5.68/4.59), right supramarginal cortex (SMC; *x, y, z* = 60, -48, 28; *t*-/*z*-values = 5.68/4.60), left middle temporal cortex (MTC; *x, y, z* = -60, -36, 0; *t*-/*z*-values = 6.69/5.13) adjacent to superior temporal sulcus (STS). For spatial WM condition, BEs compared to novices had greater FC between the left IPC seed and left lateral frontal cortex (LFC; *x, y, z* = -56, 28, 16; *t*-/*z*-values = 7.10/5.33). However, the strengths of FC between these regions had no significant correlations with behavioral performances during the task and training durations in BEs.

## Discussion

To address the question as to whether BEs, individuals having cognitive expertise including the highest level of domain-specific pattern recognition, differ from novices in untrained cognitive functions in terms of behavioral performance and brain function, here we explored the brain function of the Baduk (the game of Go) experts while performing n-back WM tasks. Despite no behavioral differences on task performance, BEs compared to novices showed greater SPC activation during face n-back task. Significant differences between BEs and novices were also found in the multifocal patterns of FC in the left FP and IPC for the face and spatial WM conditions, respectively, further showing greater functional couplings between frontal and parietal and temporal regions in BEs compared to novices.

The present study demonstrates that BEs with long-term training do not show an increase in WM ability but have disparate functional neural patterns. Consistent with our results, the same pattern of the absence of far transfer occurs in different types of training, including chess, music, and video game training (for a brief review, see Sala and Gobet, 2017a). For example, previous behavioral investigations have examined the correlates of expert performance (Chase and Simon, 1973a,b; Sala and Gobet, 2017b) and the effects of chess instruction on untrained tasks (Sala and Gobet, 2016). Some recent studies have reported the skill effect in the recall of meaningless domain-specific material (e.g., shuffled chess positions) (Gobet and Simon, 1996a,b; Sala and Gobet, 2017b) that contradicts the earlier claim for the lack of that skill effect (Chase and Simon, 1973b). However, the skill effect with meaningless material observed in experts is accounted for by meaningful chunks that occur in the position by chance, rather than superior cognitive function (Sala and Gobet, 2017b). A large number of the studies showing the effects of chess instruction on academic achievement (e.g., mathematics and literacy) suffered from the problem of confounding due to the overall poor design (e.g., the lack of control groups and no random assignment to groups; Sala and Gobet, 2016). It is thus suggested that engaging in intellectually demanding activities modifies the brain but the benefits are domain-specific rather than untrained cognitive abilities. Our findings from previous and present studies fit well with this pattern, showing differences in brain structure and function between BEs and novices, including structural connectivity (Lee et al., 2010), structural morphology and resting-state FC (Jung et al., 2013).

All BEs included in the present study have trained for 12.60 ± 1.55 years since their childhood. Achieving superior ability in one domain requires long periods of deliberate practice, which is known as a “10-year (or 10,000-h)-rule” (Ericsson et al., 1993). However, the deliberate practice is necessary but not sufficient to account for individual differences in experts and novices in music, sports, education, and board games (Campitelli and Gobet, 2011; Macnamara et al., 2014; Hambrick et al., 2018). Genetic predisposition and general intelligence may have more impact on ability than practice (Mosing et al., 2014, 2016; Sala et al., 2017). For example, more intelligent people tend to engage and excel in intellectually demanding activities such as chess (Burgoyne et al., 2016; Sala et al., 2017). In this regard, if general intelligence is controlled for (in our case, the IQ-matching of the two groups), differences between experts and novices in terms of untrained cognitive ability, WM, disappear. Our behavioral results that show no group differences support that idea. According to the theories that explain the domain-specificity of the effects of training, BEs use their knowledge structures of Baduk positions in long-term memory, called chunks and templates, as encoding and retrieval strategies, with less WM resources (Chase and Simon, 1973a; Gobet et al., 2016). However, given that the chucks (domain-specific information) are the building blocks of one’s expertise, such information is not transferable across domains. Therefore, our behavioral results may reflect that the chucks of experts are invalid for untrained tasks.

In the present study, both groups showed similar activation patterns in the frontoparietal areas and matched task performances for both face and spatial WM conditions. However, when comparing neural activity between groups, BEs compared to novice had greater SPC activation during face WM, particularly in response of the contrast of the face 1-, 2-, and 3-back versus 0-back control conditions. The *post hoc* analysis revealed that this activation difference was caused by less SPC activation in BEs, compared to novices, for the 0-back condition. The SPC is known to be involved in the top-down allocation of visual spatial attention (Giesbrecht et al., 2003; Shomstein, 2012). Given that the 0-back condition requires sustained attention/vigilance or recognition to a pre-specified target rather than WM (Owen et al., 2005) and in this condition BEs had less SPC activation, it is conceivable that visual or attention processing, rather than WM, might be different between the two groups. For example, BEs may use less attentional resource for perception and recognition of visual stimuli including face that requires at least partly holistic processing like board games, through long-term training (Guida et al., 2012, 2013). One speculated mechanism underlying the decreases of neural activity and GMV in experts is the usage-dependent possible selective elimination of synapses (Huttenlocher and Dabholkar, 1997; Takeuchi et al., 2011). Another possible explanation for our neural findings is that neural activation patterns observed do not necessarily represent training-induced changes in untrained tasks because their effects are very likely to be domain-specific. A recent longitudinal study has found no effects of commercial web-based cognitive training on brain activity and behavioral performance during decision-making as untrained tasks (Kable et al., 2017). As mentioned above, the IQ-matching of the two groups eliminates differences in untrained cognitive abilities. That is why various studies have found training-related neural patterns even in the absence of transfer effects on cognitive ability.

Expertise in board game playing may be associated with the change of FC between brain regions, rather than regional neural activity, to efficiently solve domain-specific problems (Duan et al., 2012b; Jung et al., 2013) and this may further affect the functional brain network associated with specific cognitive functions (e.g., the fronto-parietal network associated with WM). Consistent with this expectation, we found significant group differences in the multivariate patterns of FC in the left FP and IPC during the face and spatial WM, respectively, using a new data-driven multivariate approach, called MDMR-based CWAS (Shehzad et al., 2014). The *post hoc* seed-based FC analyses for these identified clusters further revealed group differences in FC between specific brain regions; BE compared to novices had greater FC between the left FP seed and several temporal and parietal areas, including the left MTC and right SMC and FFA, during face WM and greater FC between the left IPC seed and left LFC during spatial WM. Neuroimaging studies have consistently reported the co-activation of multiple frontal and parietal regions during spatial attention (LaBar et al., 1999; Ptak, 2012), WM (LaBar et al., 1999; Owen et al., 2005), and fluid reasoning tasks (Lee et al., 2006; Hampshire et al., 2011), suggesting the involvement of the frontoparietal network in such cognitive functions. A meta-analysis of functional neuroimaging studies using n-back tasks has been reported that the FP is one of regions consistently activated across all n-back studies and that has suggested that the FP plays an important role in the coordination of information processing and information transfer between multiple operations when solving the problem that requires two or more separate cognitive operations than one discrete cognitive process (Owen et al., 2005). The IPC, FFA, and MTG are higher-order visual areas. The IPC is involved in spatial perception as part of dorsal visual stream, whereas the FFA and MTG are involved in object and motion perception as part of ventral visual stream (Ungerleider and Haxby, 1994). Especially, the FFA is considered as a general visual expertise module that mediates automatic holistic processing of any higher familiar visual stimuli rather than face (Gauthier et al., 1999, 2000), while the posterior part of MTC adjacent to STS mediates object and face recognition (Hein and Knight, 2008; Bilalić et al., 2010). Recent studies using chess-related tasks for chess experts have demonstrated that both the MTC and FFA are related to object and pattern recognition for chess pieces and positions, respectively (Bilalić et al., 2010, 2011; Bilaliæ, 2016) and that the IPC is involved in an active search for patterns or chunks when processing distorted structure in their trained domain, such as random chessboards (Bartlett et al., 2013). Based on aforementioned findings and roles of these regions mentioned above in cognitive components involved in playing board games, altered FC between these regions in BEs may be associated with visual expertise acquired through long-term training. Our results may reflect the functional reorganization of BEs’ brain in a way that increases the strength of FC between frontal and parietal regions for spatial WM or adds new functional interactions between regions in the network and other regions, including FFA and MTC, for face WM that requires holistic processing.

The present study had some limitations to be addressed in future research. First, a relatively small sample size and scanning on a 1.5 T magnet may lead to resultant low statistical power and to limit the spatial resolution, respectively. However, our sample size corresponded to samples used in previous studies examining the differences in brain function between BEs and novices (Bilalić et al., 2011; Bilaliæ, 2016). Given that the neural data are not always consistent and there is currently an increasing interest in replication in psychology, future research with larger samples is needed to confirm the reliability of the present findings. Second, as a result of the cross-section nature of this study, it is unclear that brain function differences we found are directly caused by Baduk training or they are pre-existing group differences that predict whether or not a person takes up Baduk rather than a result of that training. Third, considering previous studies showing an interaction between resting-state activity and stimulus-induced activity (Northoff et al., 2010; Fransson et al., 2018) and significant differences in resting-state activity between experts and novices (Jung et al., 2013; Dong et al., 2014, 2015), it is speculated that different resting-state activity patterns between the two groups may be the foundation for the activity difference during task-state. Further research with both resting-state and task-stat fMRI will help to clarify this issue. Finally, we used the WM tasks and found neural differences between experts and novices. Thus, our findings raise some questions to be explored by future research. Do the aspects of brain function where we have identified differences are associated only with WM tasks, or are they also associated with domain-specific cognitive skills (recall of Baduk positions)? Future longitudinal studies with measure of both trained and untrained tasks are needed to address such issue.

To our knowledge, this is the first study to examine whether there were differences in the functional activity and connectivity between BEs and novices while performing standard n-back WM task with both the face and spatial WM conditions, associated with the frontoparietal network, unlike previous studies to test domain-specific pattern recognition. Despite no behavioral differences, greater SPC activation in BEs compared to novices was observed during face WM. We also found altered connectivity in the FP and IPC in BEs in terms of multivariate patterns of FC using a new data-driven multivariate FC approach and further observed greater FC between frontal and parietal and temporal regions in BEs during WM. Our results provide novel insights into the mechanism behind Baduk expertise beyond domain-specific cognitive ability and provide evidence for differences in brain circuits associated with WM ability between experts and novices.

## Author Contributions

WJ and JK designed and supervised the research. WJ, YY, and C-HC performed the experiments and analyzed data. WJ, YY, and TL wrote the manuscript. All authors reviewed the manuscript.

## Conflict of Interest Statement

The authors declare that the research was conducted in the absence of any commercial or financial relationships that could be construed as a potential conflict of interest. The reviewer VT and handling Editor declared their shared affiliation.

## Footnotes

1 http://english.Baduk.or.kr

2 http://english.Baduk.or.kr/

3 www.fil.ion.ucl.ac.uk/spm

4 http://czarrar.github.io/connectir/

5 http://marsbar.sourceforge.net/
